# Moisture Influence on Compressive Strength of Calcium Silicate Masonry Units–Experimental Assessment and Normative Calculations

**DOI:** 10.3390/ma13173817

**Published:** 2020-08-29

**Authors:** Halina Garbalińska, Jarosław Strzałkowski, Agata Stolarska

**Affiliations:** Department of Building Physics and Building Materials, Faculty of Civil Engineering and Architecture, West Pomeranian University of Technology Szczecin, al. Piastów 50, 70-311 Szczecin, Poland; Halina.Garbalinska@zut.edu.pl (H.G.); siwinska@zut.edu.pl (A.S.)

**Keywords:** calcium silicates, moisture, compressive strength, porosity structure, SEM, EDS

## Abstract

This paper primarily assesses the scale of adverse changes to the compressive strength of different types of silicates due to the influence of moisture. The study covers three groups of silicate units of different strength classes—15, 20 and 25—obtained from three different manufacturers. It was demonstrated that in all studied groups, moisture significantly decreased the compressive strength by about 30–40%. In addition, microstructural studies were conducted to analyze the relationship between the specific porosity structure of each group of silicate bricks and their compressive strength. On the basis of SEM (Scanning Electron Microscopy) and EDS (Electron Dispersive Spectroscopy) analysis, the elemental composition of individual silicates was determined and the contact zone between the aggregate and the binder was determined, which largely influenced the obtained compressive strength of each silicates. Next, the study referred to the utility of the normative procedure used to determine the strength class of samples with different geometries and at different moisture concentrations. The results of the calculations showed the high accuracy of the normative-based assessment of strength class, regardless of the manufacturer and the moisture values during examination.

## 1. Introduction

The calcium silicate brick was invented in Great Britain, and it was patented there in 1866. However, as with many other British inventions, it was first practically applied out of its borders: in Germany at the end of the 19th century. Some years later, at the start of the 20th century, the invention returned to the UK [1]. In Germany, Holland and the former Soviet Union, where sand is widely distributed, this class of brick became a main structural building material. It was so popular that a special law was introduced in Holland to protect the clay brick. At present, the calcium silicate brick is dominant in the Netherlands and Germany, and used extensively throughout Europe [1].

For example, in Polish climate conditions, the three most popular materials for wall construction are autoclaved aerated concrete, ceramics and silicates. Their respective shares of the wall material market in 2017 were 39.0%, 28.6% and 15.1%. While the share of autoclaved aerated concrete has remained near 40% for many years, the share of ceramics has dropped from 37% to 28.6% in the past decade, and the silicate share has increased from 9% to 15.1% [2].

The reason for the growing interest in silicate products is their numerous technical, health and environmental benefits, which are largely due to the use of only natural materials, such as sand (88–92%), lime (8–12%) and water (3–5%) [3]. Silicate bricks are considered to be a “green building material” and can be used as a substitute for burnt clay bricks by offering advantages for agricultural land protection, environmental protection and energy saving [4].

As is mentioned in [5], the production of clay bricks consumed huge amounts of clay in China. In the last 50 years, approximately a 1.3 billion m^2^ cultivated area has been destroyed, and about 70 million tons of coal has been consumed in the process of clay brick production.

The data given in work [6] show how many harmful substances appear in clay brick production, which contributes largely to environmental damage. A brick kiln emits 70–282 g of carbon dioxide, 0.001–0.29 g of black carbon, 0.29–5.78 g of carbon monoxide and 0.15–1.56 g of particulate matter per kilogram of clay brick, depending on the type of kiln and fuel used for the firing process. Moreover, depending on the technology, the production consumes about 0.54–3.14 MJ of specific energy per kilogram of clay brick.

Considering the negative environmental impact of the conventional brick production process, researchers and brick manufacturers all over the world have been trying to come up with alternative materials that will be more eco-friendly, but also durable and relatively cheap and have sustainability potential. Unquestionable environmental advantages result from the application of unfired bricks and the adoption of suitable stabilization techniques [7,8,9,10].

At the same time, numerous attempts are being made to partially replace clay with recycled waste materials in the production of bricks [5] (e.g., slag [11], pumice [12], waste marble [13], paper waste [14], waste glass [15]).

Having considered ecological aspects, autoclaved lime–sand brick, also known as calcium silicate brick, is a much more environmental-friendly sustainable material. Firstly, harmful substances are not emitted in its production process. Secondly, it is possible to recycle numerous waste materials by adding them into the silicate brick manufacturing process. Some researchers assess the influence of waste material addition into the silicate brick, analyzing, amongst other things, the microstructure and strength parameters [16,17,18,19,20,21,22,23].

Over the last few decades, pure calcium silicate bricks (made from sand, lime and water) have been produced. Pure silicate products are included in the safest building materials in terms of natural radioactivity [24]. They feature excellent acoustic parameters and a very good heat capacity.

They are also characterized by high resistances to both chemical and biological corrosion, and are non-combustible, so no harmful compounds will be emitted if a fire occurs. Undoubtedly, their growing popularity is also a result of their relatively high compressive strength, which is comparable to the strength of lower-class concretes. In practice, this means that this class of material can be used to construct walls in multi-storied buildings. Its relatively high strength parameters enable it to be used in structural elements that are subjected to higher loads, such as pillars, bay window corners, support areas for long lintel beams, etc.

Unfortunately, the properties of silicate products deteriorate significantly when heavy wall moisture occurs. This is especially a problem for the lower wall parts if, for example, they are exposed to flooding or groundwater, or if waterproofing is incorrectly applied or absent entirely. The technical characteristics provided by manufacturers are most often only for dry materials, while most of their parameters tend to deteriorate with increasing moisture content [25].

The problem of strength deterioration as a result of moistness has been analyzed in many articles. The researchers dealt with techniques of moisture content measurement (e.g., [26]) and assessed its impact on masonry units, mortar and wall strengths (e.g., [27]). In [28], the compressive strength of fired-clay bricks, cement mortar and hydraulic lime mortar are investigated in dry and saturated conditions. Masonry triplets (cement-based mortar) were tested in dry, moist and saturated conditions. The results were correlated with the microstructural parameters of the materials, i.e., total voids amount and pores size distribution. The fundamental mechanical properties of autoclaved sand–lime brick masonry were studied in [29], and selected properties of autoclaved bricks in [30]. The effect of sorption capacity on the mechanical properties of unfired clay bricks was investigated in [31]. The validation of selected non-destructive techniques of determining the compressive strength of masonry units was described in [32]. The researchers dealt with semi-non-destructive, non-destructive and ultrasonic methods used for autoclaved aerated concrete, in the four density classes of 400, 500, 600 and 700 kg/m^3^, tested at various moisture contents.

In particular, the influence of moisture on the strength properties of calcium silicate units was examined in [33]. The experiments were conducted on high precision calcium silicate units, which were assigned to strength class 20 and density class 2.0. The calcium silicate units were firstly adjusted to three different moisture contents: (a) oven dry, (b) 4% by weight and (c) 10% by weight. The highest mean compressive strength values were reached by the oven dry units—31.9 MPa. Related to the compressive strength in the oven dry state, the strength in the wet state amounted to between 0.67 (10% by weight) and 0.71 (4% by weight).

The problem of the negative influence of moisture concerns the majority of masonry units. Scientists from the Building Research Institute in Warsaw have recently conducted a number of experiments concerning the most popular masonry units in Poland [34]. The smallest decrease in compressive strength caused by moisture was recorded in the case of clay masonry units. This parameter has been reduced by 5–11% in relation to the dry material. A substantial percentage loss of compressive strength, caused by water saturation, occurred with autoclaved aerated concrete units, and it oscillated between 37% and 40%. By contrast, clearly the highest percentage of strength decrease was observed in the case of silicate components. The compressive strength of silicate units saturated with water showed a decrease of 42–51% relative to analogous dry products.

The problem is technically significant because, while the negative effects associated with autoclaved aerated concrete softening are widely known, it is also possible to associate silicates with relatively water-resistant materials that do not show such significant compressive strength changes. Due to the moisture resulting from the lack of waterproofing or incorrect assembly, or caused by the action of, e.g., flood waters, such changes can have a very negative impact on the safety of the building construction.

The problem of a significant deterioration in strength caused by moisture is not sufficiently highlighted in the literature—even in extensive review papers, e.g., [35].

To diagnose the extent to which moisture adversely affects the properties of silicate products, comprehensive diagnostic studies were carried out within the framework of [25]. To extend the diagnosis to include a wider range of commercially-available silicate products, the study was conducted on three groups of silicate units obtained from three different manufacturers. In a comprehensive multi-stage experiment, different basic material parameters were determined.

Another problem related to masonry is its thermal performance in fire. Harmathy et al. [36] researched the influence of moisture content in masonry walls on its fire endurance. The presence of moisture usually benefits fire endurance. In general, a 1% increase in moisture (by volume) is capable of increasing the fire endurance by 4% to 5%. However, above a certain critical level, moisture may cause explosive spalling in the case of fire. An extensive elaboration has been prepared by Naser [37,38] about the properties of common construction materials at elevated temperatures. The proposed material models have the potential to regulate and modernize structural design under extreme loading conditions, i.e., fire. The results of this investigation demonstrate the value of utilizing artificial intelligence (AI) in comprehending the impact of the complex nature of temperature-induced effects on building materials.

Manufacturers of calcium–silicate blocks ensure that these materials offer excellent acoustic insulation. This is due to their large weight. For example, in order for a wall to fulfil its acoustic requirements [39], a silicate wall with a thickness of 12 cm is sufficient. In terms of energy efficiency, silicate walls are a very advantageous solution due to their heavy weight, and thus their high heat capacity and thermal inertia.

Nurzyński in [40] searched for dependencies in the thermal and acoustic behaviours of various building elements, including silicate bricks. He showed that finding a simple relationship between acoustic and thermal insulation is quite difficult, and that massive homogeneous walls made of concrete, ceramic bricks or silicate blocks usually have good acoustic insulation and poor thermal resistance.

As noted in [41,42] the correlation between thermal resistance and sound insulation is possible for monolithic, homogenous and relatively thick walls of similar structure, but the thermal transmittance of such walls is so high that if they are used for an exterior shell they need additional insulation. Massive walls with additional lining behave, in acoustic terms, differently from bare homogeneous structures. Therefore, any correlation derived for heavy bare walls is of little practical use, when for multilayer and more complex partitions finding any correlation appears unlikely.

The results of strength studies were presented in the article [43]. They were linked to the absorbency analysis, density, and the distinct microstructures of individual silicate products, expressed by their varied pore distribution differential curves.

This article focuses on the compressive strengths of the three groups of silicates that were tested within [25]. At the beginning, all silicate specimens were exposed to water, which spread height-wise, carried up by capillary suction. Then, all specimens were cut horizontally into two pieces. The lower part was saturated with water, and the upper part was subjected to drying. Strength studies were carried out in order to discuss the two goals of the research.

The first objective concerns the influence of moisture on the reduction of the compressive strength of silicates of various strength classes. The second goal was to assess the accuracy of the normative procedures used to determine the compressive strength class of samples of different geometries and different moisture contents.

## 2. Materials and Methods

Three groups of calcium silicate products from different manufacturers were evaluated. Manufacturer names have been replaced by the symbols “E”, “X” and “B”, whereas masonry units categorized into particular groups were marked with CS-E, CS-X and CS-B symbols.

Each of these were rectangular solid units with the following dimensions [44] (*l_u_* length, *w_u_* width and *h_u_* height): CS-E: 240 × 115 × 71 mm; CS-X: 250 × 120 × 65 mm; CS-B: 240 × 120 × 80 mm, according to the manufacturer’s declaration.

Calcium silicate units, as well as other masonry units (in accordance with EN 771, Parts 1–6 [45,46,47,48,49,50]), were divided into four groups, according to the percentage, size and orientation of holes in the units when laid. Due to the fact that the masonry units chosen for the research did not feature any holes, all of them belonged to Group 1.

However, it was assumed that the particular groups of calcium silicate units CS-E, CS-X and CS-B should be differentiated via classification based on compressive strength. Calcium silicate masonry units are classified according to the normalized compressive strength classes given in EN 771-2 [46] (Annex D). The products from the manufacturers chosen for the research represented the most common compressive strength classes, 15 (CS-E), 20 (CS-X) and 25 (CS-B), with normalized compressive strengths of 15.0 N/mm^2^, 20.0 N/mm^2^ and 25.0 N/mm^2^, respectively.

Before conducting the strength tests, all samples were exposed to water which was introduced into the tested elements by capillary suction, to obtain a diverse distribution among the tested specimens. The idea was to reproduce in laboratory conditions a situation in which an element of an external wall is directly exposed to liquid water for a prolonged time.

Six units from each manufacturing plant (CS-E, CS-X and CS-B) were vertically placed in water-containing trays to orient the front surface at the suction surface. The water level was regularly replenished so that it reached a constant height of approx. 2 mm, measured from the bottom of each specimen.

The changes in the mass of individual samples were systematically recorded to determine the water sorption coefficient *A*, assigned to each of the 18 tested samples. This parameter describes the behavior of the porous material in contact with liquid water introduced into the porous material during the first period of capillary suction. It is defined in the EN ISO 9346:2007 standard [51] using the following formula:

(1) $$m_{s}=A\times \sqrt{t}$$

where *m_s_* is the specimen mass, divided by its bottom surface in contact with water, while *t* is the duration of the process. The average values of the water sorption coefficients, *A*, were then calculated.

The studies of capillary action were conducted over six weeks (about 1000 h), after which all samples were lifted from the trays and cut into halves. The upper halves were dried to a constant mass in a laboratory dryer with a gradual increase in temperature from 40 °C → 70 °C → 105 °C. In turn, the bottom halves were placed in a water bath, where they remained until they were completely saturated.

**Figure 1:**
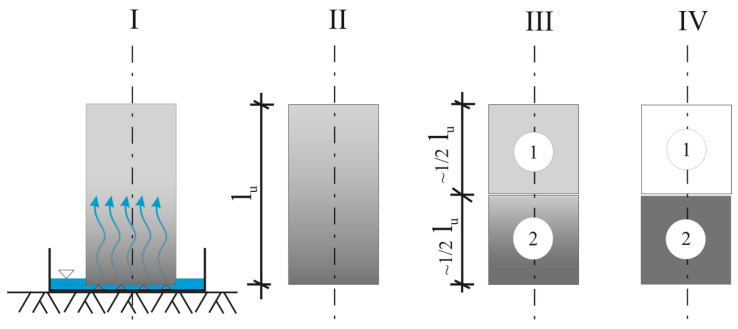
The scheme developed in-house showing the successive stages of preliminary experimental work, marked with symbols I, II, III and IV.

Figure 1 gives a schematic representation of these successive stages of preliminary experimental research: I—calcium silicate masonry unit during capillary suction testing; II—masonry unit on completion of the capillary test (varied redistribution of moisture along *l_u_*); III—masonry unit after cutting into two specimens (top (no. 1) with lower moisture content and bottom (no. 2) with a higher moisture content); IV—sample no. 1 after conditioning to the oven dry state and sample no. 2 after conditioning by immersion in water bath.

A total of 18 samples in the dried state (6 upper halves of each series) and a total of 18 samples in the saturated state (6 lower halves of each series) were used for strength tests. The load-bearing surfaces were not subjected to additional treatment because they were flat and even. The processes of specimen preparation and their numbering were in accordance with EN 772-1 [52]. Furthermore, the placing of specimens in the testing machine, as well as the settings of the loading rate, were in accordance with the recommendations included in the EN 772-1 standard [52].

First, compressive strength values were determined for all specimens no. 1 (dried to a constant mass), and then for all specimens no. 2 (conditioned by water immersion).

The maximum load obtained for each specimen was recorded, and then its compressive strength was calculated by dividing the measured compressive force value (N) by the load-bearing area (mm^2^). In all cases the gross area of the loaded surface was calculated in square millimeters by multiplying the length (≈½ *l_u_*) by the width (*w_u_*) of each specimen determined in accordance with EN 772-16 [44].

The obtained values from the compressive strength tests are summarized in tabular form in [43]. In each of the 36 samples (18 dry and 18 saturated), the determined value of the compression strength *f_c_* rounded to 0.1 N/mm^2^ was given. Based on these values, graphic comparisons of the results were prepared.

## 3. Results and Discussion

### 3.1. Results of Strength Tests in Terms of Moisture and Microstructure Effect

**Figure 2:**
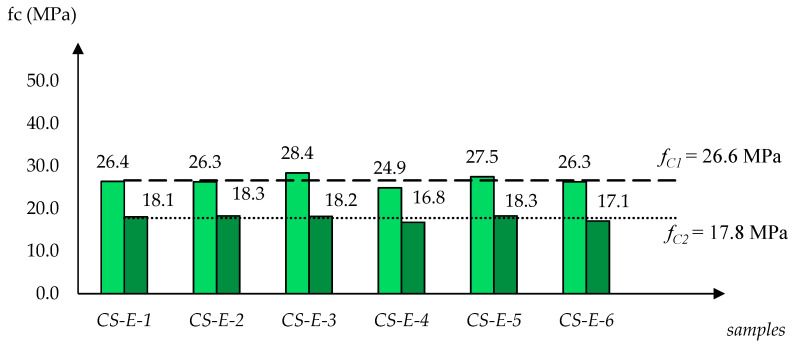
Overview of compressive strength values designated for silicate specimens CS-E-1–CS-E-6 (6 dry and 6 water-saturated), with *f_C1_* (for dry state) and *f_C2_* (for saturated state) averages.

**Figure 3:**
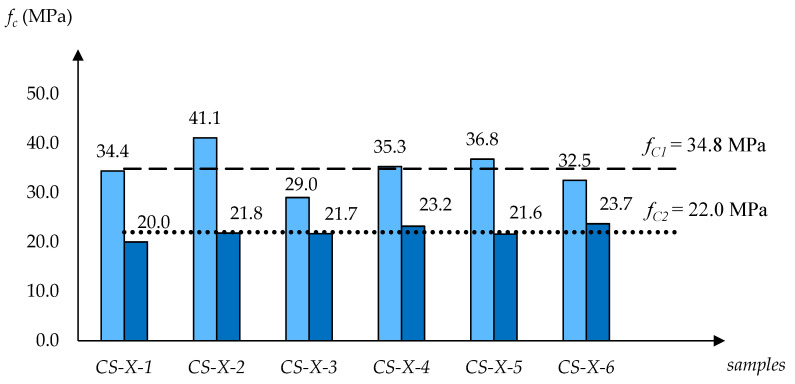
Overview of compressive strength values designated for silicate specimens CS-X-1–CS-X-6 (6 dry and 6 water-saturated), with *f_C1_* (for dry state) and *f_C2_* (for saturated state) averages.

**Figure 4:**
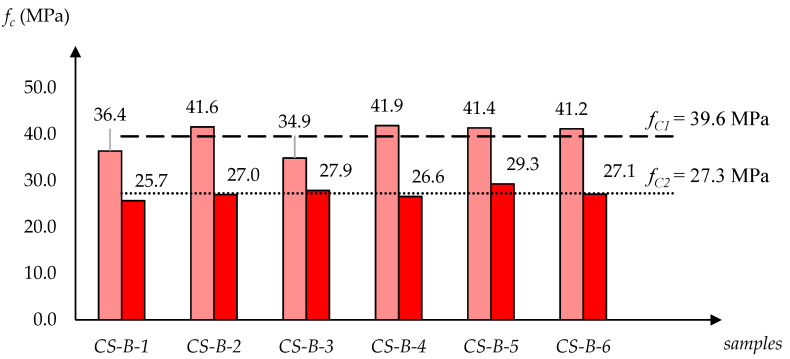
Overview of compressive strength values designated for silicate specimens CS-B-1–CS-B-6 (6 dry and 6 water-saturated), with *f_C1_* (for dry state) and *f_C2_* (for saturated state) averages.

Figure 2, Figure 3 and Figure 4 show how the determined strength values of the specimens varied between the dry and water-saturated states. The specimens are grouped by producer and, to avoid presenting manufacturers’ names, they were described with symbols: CS-E, CS-X and CS-B. In each case, the strength values are quoted for two moisture states; firstly for six specimens dried to a constant mass, and then for six water-saturated specimens. Additionally, in Figure 2, Figure 3 and Figure 4 the average compressive strength values for the dry state (*f_C1_*) and for the saturated state (*f_C2_*) are shown.

A clear tendency of compression strength decrease was observed as a result of water saturation. In case of the CS-E group of silicates the drop *f_C2_*/*f_C1_* was 66.9%, and in the case of groups CS-X and CS-B, the drops were 63.2% and 68.9%, respectively.

As noted in [43], this is an issue of great technical importance, since the most significant deterioration of mechanical parameters due to strong moisture occurs mainly in the lower parts of the wall exposed to groundwater or floods. The lower parts of the masonry construction carry a much greater load than the walls of higher floors, and are therefore subjected to much higher stress levels, and the moisture-induced strength decrease can jeopardize the safety of the entire structure.

It is commonly accepted that the properties of a porous material (including strength parameters) are determined by its internal structure—the skeletal structure and specificity of pores, particularly those that can be filled with water in the liquid phase.

For this reason, in the compilation in Table 1, additional data characterizing the tested silicates were included. For the three groups CS-E, CS-X and CS-B, the average compressive strength values in the dry state *f_C1_* and in the saturated state *f_C2_*, and the water sorption coefficients, *A,* are shown in Table 1. The average density in the dry state, *ρ_p_*, and average water absorption by weight, *n_w_*, and volume, *n_v_*, were obtained from [25,43], which provides detailed data for all the studied silicates.

An analysis of the data collected shows that the CS-E and CS-B products have similar water absorptions by weight *n_w_* (13.4% and 14.3%) and volume *n_v_* (24.7% and 26.1%), as well as similar apparent densities *ρ_p_* (1.840 kg/dm^3^ and 1.830 kg/dm^3^). However, their compressive strength parameters are significantly different. While the CS-E products have the lowest compressive strength values, the CS-B products exhibit the highest parameters in this respect. For CS-E, CS-X and CS-B, the values of *f_C1_* (in the dry state) and *f_C2_* (in the saturated state) are the following: *f_C1_* = 26.6 MPa < 34.9 MPa < 39.6 MPa and *f_C2_* = 17.8 MPa < 22.0 MPa < 27.3 MPa. Similar trends were found in the case of the sorption coefficient, *A* = 3.6 < 5.2 < 8.3 kg/m^2^h^0.5^. It should be noted, however, that the highly different *A* values also have no direct connection to the other physical parameters (*ρ_p_*, *n_w_*, *n_v_*) shown in Table 1.

Therefore, it can be concluded that the macroscopically-determined physical parameters of the materials (density, water absorption and water sorption coefficient) do not provide useful information for drawing conclusions regarding the potential compressive strength of given material groups.

Therefore, additional microstructural studies were carried out using mercury porosimetry and scanning electron microscopy (SEM), to clarify the differences in the porosity structures of individual groups of silicates.

**Figure 5:**
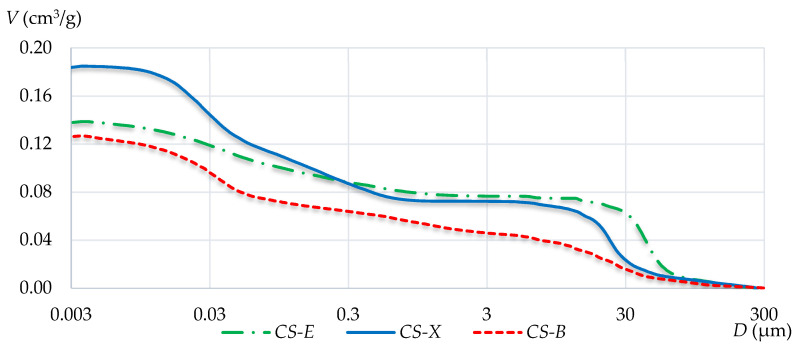
Integral pore distribution curves of the three tested silicates.

**Figure 6:**
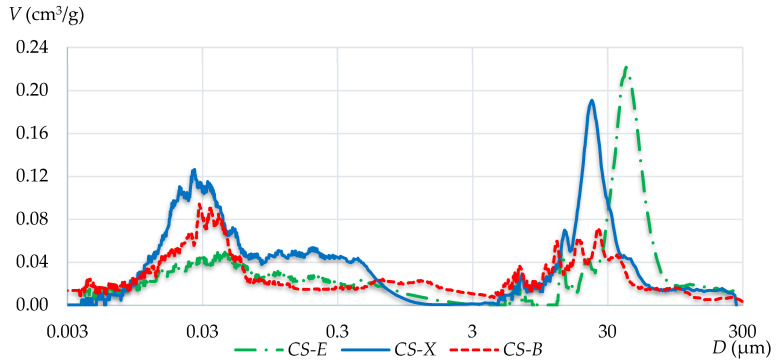
Differential pore distribution curves of the three tested silicates.

Figure 5 and Figure 6 show integral and differential curves, which respectively represent the pore distribution.

The integral curves for CS-E and CS-X silicates show a significant increase in porosity at a pore diameter of about 30 µm. Such a situation does not occur in the case of the CS-B silicate, which generally has a more homogeneous porosity distribution. By analyzing the CS-B integral curve, a more pronounced deflection in its course can be observed in the pore diameter region of about 0.05 µm.

Differences in the distribution of pores can be assessed more clearly on the basis of differential curves assigned to individual materials. These curves are all characterized by the bimodal pore distribution of the tested silicates. Two areas with increased pore contents, but with a different proportion of pores, are clearly visible on all differential graphs. For CS-E and CS-X specimens, there are clear porosity peaks with diameters of 42 µm and 23 µm, respectively. For CS-B silicate, no dominant diameters are observed in this range. The pores of more than 3 µm in diameter are more evenly distributed, and their contents are maintained at significantly lower levels than in the CS-E and CS-X silicates. The second concentration range of pores appears between 0.01 and 0.07 µm. The highest amount of pores in this range of diameters is found in the CS-X silicate, and it is slightly lower in the CS-B silicate. The CS-E silicate shows clearly the lowest content of these small pores. No dominant diameter has shown its presence in any case, and the concentration range always extends with a wider spectrum of pores.

Based on the determined values and the tested relationships, it can be stated that density and water absorption do not provide useful information for predicting potential strength characteristics. It was shown that it is not so much the total volume of pores but rather their specific geometry, determined by additional tests, that is the most important. The analysis of the obtained integral and differential pore distribution curves indicates the different porosity characteristics of each of the tested silicates. Even if a material contains many pores, if they have small diameters, the material will possess relatively high strength parameters. Therefore, large capillary pores and air voids have the most negative impact on the strength characteristics. A comparative analysis of the integral curves shows that the CS-E silicate units contain the largest fraction of pores in the range from 0.3 μm to 300 μm. In CS-X, this value is lower, while CS-B clearly shows the lowest amount of such pores. This is also confirmed by the differential curve analysis, which indicates that the CS-E silicate brick has the largest amount of pores that range from 3 μm to 300 μm, compared with CS-X and CS-B. The last two, in turn, demonstrated the highest share in terms of the smallest pore content that did not negatively affect the strength. The determined specific redistribution of the largest pores was shown to have a direct impact on the macroscopic strength relationships, in the order *f_CS-E_* < *f_CS-X_* < *f_CS-B_*.

**Figure 7:**
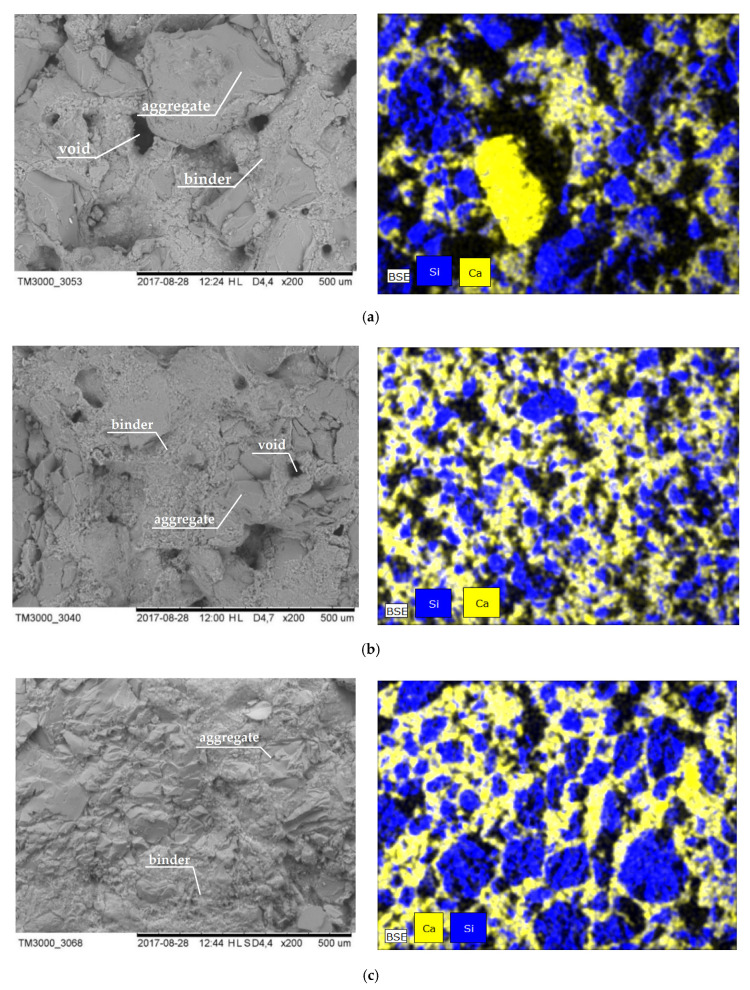
SEM (Scanning Electron Microscopy) images (**left**) of individual silicate samples at ×200 magnification: (**a**) CS-E, (**b**) CS-X, (**c**) CS-B and the EDS (Electron Dispersive Spectroscopy) results (**right**).

Figure 7 shows the SEM images of three silicates at ×200 magnification. They depict the quantitative fractions of large pores (responsible for decreased strength) within the second peak, covering the range from 3 to 300 μm.

The scanning electron microscopy (SEM) images in Figure 7 confirm the results obtained using mercury porosimetry. Photos a) and b) for, CS-E and CS-X respectively, show a large number of pores with diameters ranging from about 5 to 50 µm, which are not visible at all in photo c) for the CS-B silicate.

The figures on the right present the EDS results, showing only the silicon, representing the aggregate location and the calcium as the binder. In the case of CS-E, numerous voids are visible, which are most likely responsible for reducing the strength of this material. The binder is not evenly distributed.

In the specimen CS-X, the used aggregate is definitely of smaller diameter than the CS-B specimens. In CS-X, the binder content also represents a greater share than that in CS-B. The CS-B specimen has certainly the smallest share of visible voids between the grains of the aggregate and the calcium binder. This translated into a higher compressive strength compared to the remaining specimens.

In the Table 2, the proportions of the weights of the main elements are presented for the tested types of silicates. The differences in proportions do not appear to be large. However, it is clearly visible that the most silicon occurs in CS-B, and the least in CS-E, which exactly corresponds to the strength classes. This shows that the most silicon-based aggregate was in CS-B, hence its greater compressive strength.

In the case of calcium, the highest value was observed in CS-X, which is confirmed by the EDS photos in Figure 7. In the case of CS-B, the amount of calcium (and thus the binder) is the lowest. This, too, may result in the increased strength of this type of silicate compared to the others.

### 3.2. Results of Strength Tests in the Context of Normative Regulations

Masonry walls are composed of units that must meet the requirements contained in the documents of PN-EN 771-1–PN-EN 771-6 [45,46,47,48,49,50]. Their subsequent parts involve clay masonry units, calcium silicate masonry units, aggregate and autoclaved aerated concrete masonry units, and manufactured and natural stone masonry units. The second part of this regulation, PN-EN 771-2 [46], describes the properties of calcium silicate masonry units.

Eurocodes have set unified methods for testing the strength of masonry units. The PN-EN 772-1 [45] recommends that the average compressive strength of masonry units be determined and then recalculated to obtain a normalized compressive strength, using the formula [53]:

*f_b_* = *η_w_ d f_C_* (2)

where *f_b_*—normalized compressive strength—is the compressive strength of masonry units converted into the air dry compressive strength of an equivalent 100 mm wide and 100 mm high masonry unit (MPa); *f_C_*—average compressive strength—is the arithmetic mean of the compressive strength values of particular specimens (MPa); *η_w_*—is the coefficient that includes the moisture state of the masonry units (using the following values: *η_w_* = 1.0 for specimens conditioned to air dry condition and conditioned to 6% moisture content, *η_w_* = 0.8 for specimens conditioned to the oven dry condition, and *η_w_* = 1.2 for specimens conditioned by immersion in water); and *d*—is the shape factor that considers the impact of the “scale effect” of tested masonry units.

Table 3 shows the values of the shape factor *d* taken from EN 772-1 [52] (Annex A), whereby the width and height of specimens should be determined in accordance with EN 772-16 [44].

Thus, the specimen’s compressive strength depends on its size, relative proportions and moisture content. For construction calculations, the normalized compressive strength *f_b_* was used. Formula (2) has been applied to establish the *f_b_* value. In accordance with the procedure described above, the average compressive strength is first converted to an equivalent compressive strength relevant to the air-dry conditioning regime. In order to obtain the normalized strength *f_b_*, the air-dry compressive strength is multiplied by a shape factor *d*, given in Table 3.

The procedure in Formula (2) was applied in [25] in relation to the results obtained with respect to the CS-E, CS-X and CS-B silicates.

The compressive strength *f_C_* for each of the tested materials was calculated as the average strength of individual specimens obtained from each manufacturer. Then, to standardize the compressive strength values, the average *f_C_* values obtained for individual groups of specimens were multiplied by the shape factor *d* and the moisture content coefficient *η_w_*.

The particular results are given in Table 4, Table 5 and Table 6 for the CS-E, CS-X and CS-B silicates, respectively. The obtained results for the normalized compressive strength of specimens in dry and wet conditions have similar values. For all three types of silicates, the results obtained confirm the classes given by the manufacturers. The coefficients *η_w_* have properly accounted for the effect of moisture on compressive strength. The proposed formula for normalized strength allows the product class to be determined with high accuracy.

## 4. Conclusions

The results of the studies carried out on samples in dry and saturated states show a clear trend whereby the compressive strength decreases as the result of the water saturation of the tested silicates. The determined variability ranges for individual products are as follows: CS-E—*f_C1(dry)_* = 26.6 MPa → *f_C2(wet)_* = 17.8 MPa; CS-X—*f_C1(dry)_* = 34.8 MPa → *f_C2(wet)_* = 22.0 MPa; and CS-B—*f_C1(dry)_* = 39.6 MPa → *f_C2(wet)_* = 27.3 MPa. The obtained quantitative relationships *f_C2(wet)_*/*f_C1(dry)_* in the case of each material were CS-E—0.67, CS-X—0.63, and CS-B—0.69.

The collected results indicate a significant percentage decrease for each of the studied strength classes. In the cases of the CS-E (strength class 15), CS-X (strength class 20) and CS-B (strength class 25) silicate units, the strength reductions exceeded 30%.

The goal of this article was to assess the physical and microstructural conditions of silicate products, and also to discuss the compatibility of normative procedures for determining the strength class of various kinds of silicates in differently-conditioned specimens. A comparative analysis of the results (Table 4, Table 5 and Table 6) shows that for all products, a very high correspondence of the data to the class provided by each of the manufacturers was obtained. Thus:

- For CS-E units, a strength class of 15 was determined for both dry (*f_b1_* = 18.3 MPa) and water-saturated (*f_b2_* = 18.4 MPa) samples;
- For CS-X units, a strength class of 20 was determined, both in dry (*f_b1_* = 22.6 MPa) and water-saturated samples (*f_b2_* = 21.4 MPa);
- For CS-B units, a strength class of 25 was determined based on both dry (*f_b1_* = 27.5 MPa) and water-saturated samples (*f_b2_* = 28.5 MPa).

Thus, it can be assumed that the proposed formula for normalized strength allows the product class to be determined with high accuracy, while considering the actual size of the tested samples and their specific moisture contents.

However, taking into account the fact that a strength drop of over 30% was noted for all tested types of silicates, every effort should be made to protect the masonry elements against moisture. This applies both to the stage of designing building components (checking the requirements of internal and surface condensation) and the stage of erecting the building (the application of properly selected and properly made waterproofing).

## Figures and Tables

**Table 1:** Comparison of the average values of the main parameters characterizing individual groups of tested silicate products.

Product Name	*f_C1_* (MPa)	*f_C2_* (MPa)	*A* (kg/(m^2^·h^0.5^)	*ρ_p_* (kg/m^3^)	*n_w_* (%)	*n_v_* (%)
CS-E	26.6	17.8	3.6	1840	13.4	24.7
CS-X	34.9	22.0	5.2	1638	18.8	30.7
CS-B	39.6	27.3	8.3	1830	14.3	26.1

**Table 2:** The proportions of the weights of main elements in the tested specimens.

Element	Element Weight (%)		
	CS-E	CS-X	CS-B
O	58.16	56.05	56.15
Si	25.82	26.31	28.47
Ca	14.87	15.66	14.15
Al	0.88	1.12	0.86
Fe	0.27	0.86	0.37
Total (%)	100.00		

**Table 3:** The values of the shape factor *d,* [52].

Height (mm)	Width (mm)				
	50	100	150	200	≥250
40	0.80	0.70	-	-	-
50	0.85	0.75	0.70	-	-
65	0.95	0.85	0.75	0.70	0.65
100	1.15	1.00	0.90	0.80	0.75
150	1.30	1.20	1.10	1.00	0.95
200	1.45	1.35	1.25	1.15	1.10
≥250	1.55	1.45	1.35	1.25	1.15

**Table 4:** The results obtained from the compressive strength tests carried out on the CS-E specimens used to convert to the strength class of the product.

Sample	*f*_c1_ (MPa)	*f*_c2_ (MPa)	*η_w1_*	*η_w2_*	$\mathrm{Averaged}\;h_{av,\;1}\;(\mathrm{mm})$	$\mathrm{Averaged}\;h_{av,\;2}\;(\mathrm{mm})$	*d* (Interpolation)	*f_b1_ = η_w_df_C1_* (MPa)	*f_b2_ = η_w_df_C2_* (MPa)	Class
CS-E-1	26.4	18.1	0.8	1.2	71	115	0.86	18.3	18.4	15
CS-E-2	26.3	18.3								
CS-E-3	28.4	18.2								
CS-E-4	24.9	16.8								
CS-E-5	27.5	18.3								
CS-E-6	26.3	17.1								

**Table 5:** The results obtained from the compressive strength tests carried out on the CS-X specimens used to convert to the strength class of the product.

Sample	*f*_c1_ (MPa)	*f*_c2_ (MPa)	*η_w1_*	*η_w2_*	$\mathrm{Averaged}\;h_{av,\;1}\;(\mathrm{mm})$	$\mathrm{Averaged}\;h_{av,\;2}\;(\mathrm{mm})$	*d* (Interpolation)	*f_b1_ = η_w_df_C1_* (MPa)	*f_b2_ = η_w_df_C2_* (MPa)	Class
CS-X-1	34.4	20.0	0.8	1.2	64	120	0.81	22.6	21.4	20
CS-X-2	41.4	21.8								
CS-X-3	29.0	21.7								
CS-X-4	35.3	23.2								
CS-X-5	36.8	21.6								
CS-X-6	32.5	23.7								

**Table 6:** The results obtained from the compressive strength tests carried out on the CS-B specimens used to convert to the strength class of the product.

Sample	*f*_c1_ (MPa)	*f*_c2_ (MPa)	*η_w1_*	*η_w2_*	$\mathrm{Averaged}\;h_{av,\;1}\;(\mathrm{mm})$	$\mathrm{Averaged}\;h_{av,\;2}\;(\mathrm{mm})$	*d* (Interpolation)	*f_b1_ = η_w_df_C1_* (MPa)	*f_b2_ = η_w_df_C2_* (MPa)	Class
CS-B-1	36.4	25.7	0.8	1.2	79	120	0.87	27.6	28.5	25
CS-B-2	41.6	27.0								
CS-B-3	34.9	27.9								
CS-B-4	41.9	26.6								
CS-B-5	41.4	29.3								
CS-B-6	41.2	27.1								
